# CNS Malformations in the Newborn

**DOI:** 10.1186/s40748-021-00136-4

**Published:** 2022-01-17

**Authors:** Kristin Barañano, Irina Burd

**Affiliations:** 1 Department of Neurology, Johns Hopkins University School of Medicine, Baltimore, Maryland USA; 2 Department of Gynecology and Obstetrics, Johns Hopkins University School of Medicine, Baltimore, Maryland USA

**Keywords:** Brain malformation, Agenesis corpus callosum, Ventriculomegaly, Dandy Walker malformation

## Abstract

Structural brain anomalies are relatively common and may be detected either prenatally or postnatally. Brain malformations can be characterized based on the developmental processes that have been perturbed, either by environmental, infectious, disruptive or genetic causes. Fetuses and neonates with brain malformations should be thoroughly surveilled for potential other anomalies, and depending on the nature of the brain malformation, may require additional investigations such as genetic testing, ophthalmological examinations, cardiorespiratory monitoring, and screening laboratory studies.

## Introduction

Structural brain anomalies have a prevalence at birth of roughly 1-2 per thousand births [1, 2]. Major brain structures can be identified via ultrasound by the end of the first trimester, enabling early diagnosis of major defects such as acrania, anencephaly and holoprosencephaly. As brain development proceeds, some defects of brain growth and differentiation can be detected during routine second trimester anatomy scans, although ultrasound alone may have limitations. Detecting such a finding would typically prompt a follow-up fetal MRI to further delineate the findings in more detail, as well as screening for TORCH infections (*t*oxoplasmosis, *o*ther, *r*ubella, *c*ytomegalovirus and *h*erpes simplex) and potentially genetic testing. These investigations may permit anticipation of a neonate’s medical needs in the first days, weeks or months of life.

**Fig. 1:**
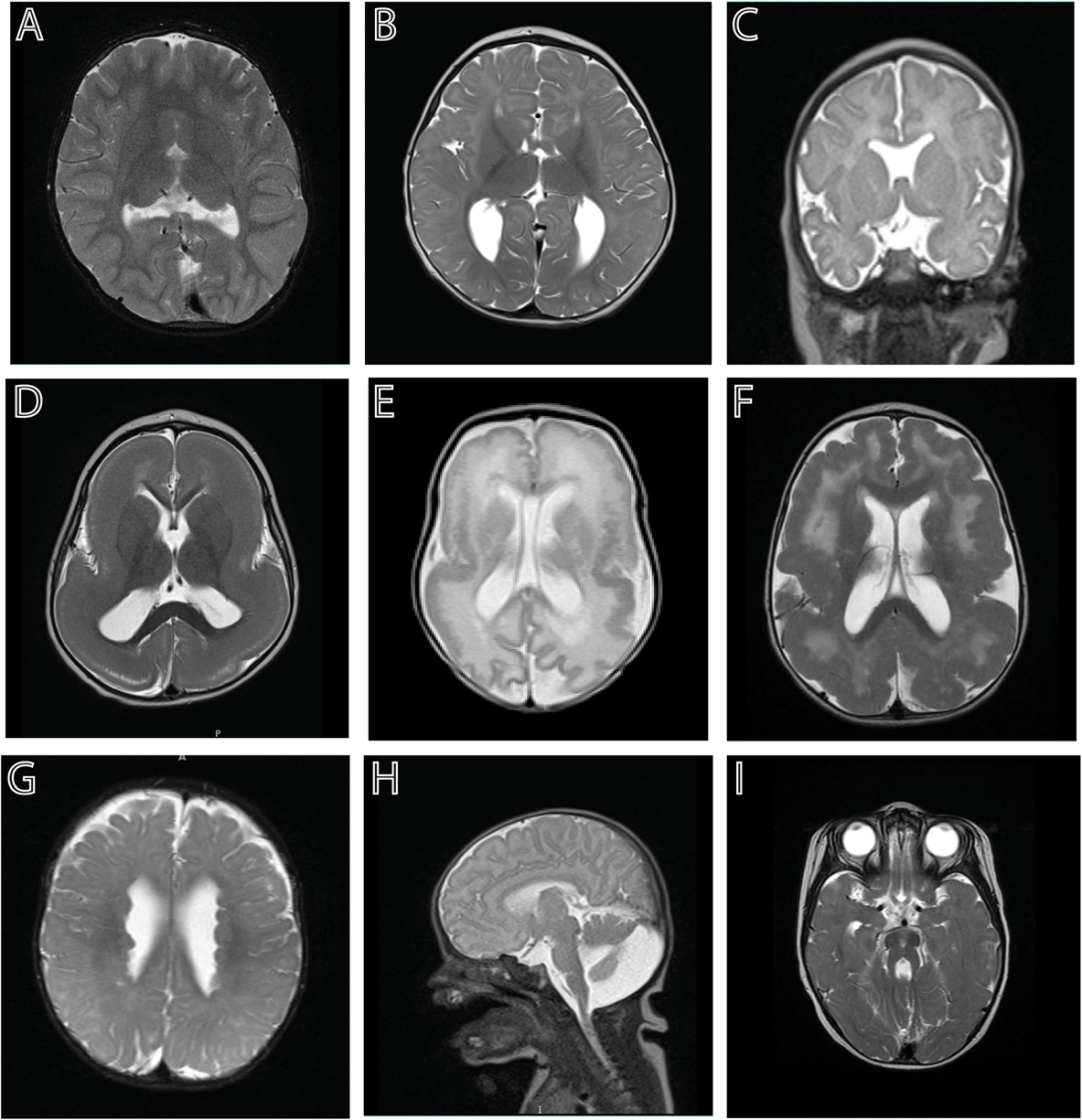
Examples of brain malformations in neonates: **A**. Semilobar holoprosencephaly; **B**. Agenesis of the corpus callosum; **C**. Absent septum pellucidum; **D**. Classical lissencephaly; **E**. CMV-associated polymicrogyria, pachygyria and white matter abnormalities; **F**. Diffuse polymicrogyria; **G**. Subependymal heterotopias; **H**. Dandy Walker malformation; and **I**. Molar tooth sign associated with Joubert syndrome

Give the limitations of prenatal ultrasound, not all brain malformations are able to be detected prenatally. A neonate with a congenital brain malformation may come to attention through a variety of other avenues. These infants may have an obvious neurological impairment, such as significant hypotonia or feeding issues, or present with clinical seizure activity. Brain malformations may also be detected incidentally with routine head imaging, such as screening ultrasounds for premature infants at risk for intraventricular hemorrhage.

In this review, we will discuss the various types of brain malformations which may be routinely encountered in the newborn period and outline suggested management strategies.

## Pathophysiology/Clinical Features

The development of the central nervous system occurs through series of major processes, including neural tube formation, prosencephalic development, neuroblast proliferation and migration, cortical organization and myelination [3]. The nature of a malformation will depend on the timing of when the various processes are disrupted. However, many genetic disorders and teratogenic exposures including TORCH infections may span over an extended time during the gestation, leading to the co-existence of multiple malformations, for example, cortical migrational anomalies in association with agenesis of the corpus callosum. 

## Holoprosencephaly

Holoprosencephaly (HPE) occurs when there is a defect in ventral induction and patterning resulting in a failure of appropriate cleavage of the prosencephalon into the deep structures and hemispheres of the brain (Fig. 1A) [4]. There is a spectrum of severity. From most to least severe, these are called alobar, semilobar and lobar HPE, and there is also a milder middle interhemispheric variant. HPE is commonly associated with chromosomal anomalies (i.e., Trisomy 13) and can be associated with recognized syndromes. Non-syndromic HPE can be associated with a number of genes in the various signaling pathways involved in this process. Defects in cholesterol metabolism are associated with approximately 10% of cases of HPE [5]. Maternal diabetes and other teratogens are considered risk factors. HPE is commonly associated with hydrocephalus and pituitary dysfunction.

## Agenesis of the corpus callosum (ACC)

The corpus callosum is a brain structure that developed relatively lately from an evolutionary perspective. It is the major neural pathway that connects the left and right cerebral hemispheres. It is only present in placental mammals, and not in marsupials or monotremes [6]. During development, pioneer axons cross the midline around 13-14 weeks of development, and by 17 weeks, all four major parts (rostrum, genu, corpus and splenium) are present. Around this time, anomalies in the corpus callosum development can typically be first detected on fetal ultrasound, either by absence of the cavum septum pellucidum, anomalous flow in the pericallosal artery or by an unusual parallel configuration of the lateral ventricles [7]. Congenital structural abnormalities include: agenesis, with either complete or partial absence of this structure (Fig. 1B); hypoplasia, referring to a corpus callosum that is thinner than expected; and dysgenesis, which results when the corpus callosum is present but malformed in some way. Syndromes with ACC can be broadly classified by the stage in development that is primarily affected. It can occur in association with disorders of neuronal and/or glial proliferation, neuronal migration and/or specification, midline patterning, axonal growth and/or guidance, and post-guidance development [8].

ACC can be an isolated, incidental finding, but chromosomal anomalies are commonly associated with ACC [9]. ACC is associated with > 100 described syndromes in Online Mendelian Inheritance in Man (OMIM) and there is also an increased risk of copy number variants associated with this [10]. ACC is frequently found in association with other anomalies, which may not be fully appreciated until fetal or postnatal MRI is performed [11].

## Septo-optic dysplasia

Septo-optic dysplasia (SOD), which also goes by the eponym of de Morsier syndrome, is hypothesized to represent a vascular disruption sequence involving the anterior cerebral artery during a critical period of neuroembryogenesis (Fig. 1C) [12]. It is defined by the presence of at least two of the three: optic nerve hypoplasia, pituitary abnormalities and agenesis of the corpus callosum or absent septum pellucidum. A few genes have been found to account for 1-2% of the cases of SOD (*HESX1*, *OTX2*, *SOX2* and *SOX3*) [13].

## Ventriculomegaly and hydrocephalus:

Ventriculomegaly (VM) is a common prenatal finding. The normal diameter of the lateral ventricles, regardless of gestational age, is less than 10 mm. VM is defined as mild (10-12 mm), moderate (13-15 mm) or severe (>15 mm) based on the size of the ventricles as measured at the level of the atria [2]. VM can be unilateral or bilateral, and asymmetry is common. It is commonly associated with chromosomal anomalies or TORCH infections, so this finding typically results in evaluation for these possibilities. The presence of VM does not invariably suggest that a fetus or infant has hydrocephalus, meaning that the ventricular system, as it drains from lateral to third to fourth ventricle and then exits into the subarachnoid space, is under pressure. When hydrocephalus is present, it is not uncommonly associated with aqueductal stenosis, which can be secondary to a mechanical blockage such as a web, or genetic in nature, such as related to pathogenic variants in the *L1CAM *gene [14]. Once ventriculomegaly is prenatally diagnosed, it is important to thoroughly examine other structures within CNS and set up prenatal follow up MRI and ultrasounds. In cases of aqueductal stenosis, it is important to know the sex of the fetus in counseling in regard to a possible X-linked genetic condition.

When VM is the only finding on prenatal ultrasound, fetal MRI reveals additional malformations about 10% of the time, which may influence counseling about prognosis [15]. Follow up prenatal ultrasounds are important in measuring fetal head parameters for determination of proper route and timing of delivery.

## Migrational disorders: lissencephaly, polymicrogyria and heterotopias

During prenatal development, neuroblasts lining the ventricular zone proliferate, and after their final mitosis, will exit the cell cycle and migrate to the cortical plate in a choreographed “inside out” migrational pattern, to form the organized six layers of the cerebral cortex [16]. When this process goes awry early on, lissencephaly (Fig. 1D) or pachygyria (Fig. 1E) can be the result, with a thickened and smoothened cortex. When migration is disrupted later in development, it can result in polymicrogyria (PMG) (Fig. 1E and F), which is a spectrum of cortical malformations with the common features of excessive gyration and microscopic abnormality of cortical structure and lamination. PMG overlaps with the cobblestone malformations like Walker-Warburg syndrome resulting from mutations in basement membrane proteins. PMG is commonly seen as a result of a disruption, such as lining a schizencephalic cleft resulting from a vascular insult or clot formation (as in cases of trauma or Neonatal Alloimmune Thrombocyotpenia). Heterotopias are abnormal accumulations of neurons in the periventricular region as a result of their failure to migrate (Fig. 1G) [17]. They are classically associated with filamin mutations in females, but are also associated with mutations in *ARFGEF2* (in association with severe microcephaly) and with a number of other syndromes and copy number variations [18]. Migrational neuronal pattern disorders (disorders of cortical development) are now recognizable with prenatal ultrasound [19].

## Cerebellar anomalies: Dandy Walker malformation and Joubert syndrome

Cerebellar anomalies affect 1:5000 live births and are frequently associated with additional CNS and non-CNS anomalies [20]. Congenital anomalies may result from developmental or genetic causes or acquired or disruptive causes such as hemorrhage, ischemia or infection. The vermis (midline) is frequently affected; predominant involvement of the cerebellar hemispheres is uncommon and suggestive of pontocerebellar hypoplasia or disruption in very premature infants [21].

Dandy Walker malformation (DWM) is the most commonly diagnosed anomaly (Fig. 1H). However, it can be difficult to distinguish a true DWM from the entities of mega cisterna magna, Blake pouch cyst and vermian hypoplasia on prenatal ultrasound, and it often requires fetal or postnatal MRI to confirm the diagnosis [22]. Diagnostic criteria for DWM include: complete or partial agenesis of the cerebellar vermis, cystic dilation of the fourth ventricle, and enlargement of the posterior fossa with elevation of the torcula [22]. With DWM, there are associated malformations, generally of the CNS, in 29-49% of individuals, and 10-17% have agenesis or dysgenesis of the corpus callosum [23]. There is an increased frequency of congenital heart disease, cleft lip and/or palate and neural tube defects. DWM can be associated with chromosomal anomalies, teratogen exposures or related to sporadic associations, such as the PHACE syndrome (posterior fossa brain malformation, hemangiomas of the face, arterial anomalies, cardiac defects, eye abnormalities) [23]. Patients may have associated hypotonia and motor delays. Cerebellar signs such as ataxia, nystagmus, tremor and dysmetria may not necessarily be seen. Children with DWM go on to require CSF shunting about half of the time.

Joubert syndrome is part of a spectrum of what are referred to as ciliopathies, as the affected gene products are components of primary cilia. Joubert syndrome and related disorders (JSRD) includes a spectrum of disorders including oral-facial-digital (OFD) and Meckel Gruber syndrome. Classical Joubert syndrome has the “molar tooth” sign (Fig. 1I) with elongation of the superior cerebellar peduncles, which are uncrossed, and a deep interpeduncular fossa. Macrocerebellum has also been described in association with JSRD [24]. Supratentorial involvement is found in 30% of patients including callosal dysgenesis, cephalocoeles, hippocampal malrotation and ventriculomegaly [25]. Early in infancy, abnormal eye movements and nystagmus may be appreciated, but oculomotor apraxia becomes apparent later. They may also have notable irregular breathing patterns. Systemic involvement can include ocular (retinal dystrophy, colobomas), renal (nephronophthisis), hepatic (congenital hepatic fibrosis) and skeletal (polydactyly).

### Evaluation and management

#### Growth parameters and vital signs

Standard parameters should be measured and monitored

#### General examination

A careful general examination is crucial. The focus should be on assessing for any dysmorphisms or associated congenital anomalies that may be suggestive of an underlying syndrome; for example, the towering forehead typically associated with Zellweger syndrome, characteristic facies of Miller-Dieker associated lissencephaly, or hypotelorism and possible nasal anomalies associated with holoprosenecephaly. The fullness and size of the anterior fontanelle should be assessed, as a large fontanelle can be associated with disorders such as Zellweger syndrome and can help with monitoring for the possible development of symptomatic hydrocephalus. 2-3 toe syndactyly can be a clue as a disorder of cholesterol metabolism such as Smith-Lemli-Opitz syndrome. Hypopigmented macules may be a clue to the presence of tuberous sclerosis-associated cortical tubers or subependymal nodules. Lines of Blashko may lead to suspicion of a mosaic genetic condition. The gluteal region should be carefully examined for any dimples or unusual clefting patterns that may suggest the possibility of an underlying spinal dysraphisms or spinal cord tethering. Arthrogryposis, or limited range of movements of limbs with contractures, can sometime be seen in association with brain malformations. Involvement of the pituitary axis can result in micropenis.

#### Neurological examination

A standard neonatal neurological examination should be performed on all infants with suspected or known CNS malformations. Such infants may have abnormalities of eye movements, or impairment of their suck and swallow. Hypotonia is common, and central hypotonia versus peripheral hypotonia related to frank weakness should be delineated.

#### Postnatal CNS imaging

Infants with a suspected CNS malformation will ideally undergo MR imaging in the neonatal period, under a standard neonatal protocol that minimizes the need for sedation. In many cases, this will simply confirm or refine the details of the CNS malformation [26]. There is some incidence of detecting additional meaningful anomalies on postnatal MRI [27], especially when prenatal imaging was limited to second trimester studies. Conversely, some fetal findings may no longer be appreciated postnatally; for example, posterior fossa findings such as vermian hypoplasia may no longer be apparent by term [28]. Such imaging, in addition to surveilling for potential additional anomalies that may influence management or anticipatory guidance, may also serve as a baseline for further subsequent imaging, such as monitoring for the development of hydrocephalus in the setting of DWM.

Some thought should be given to ultrasound of the spine or including spine on MR imaging, given the risk of a tethered cord as an additional anomaly, in particular for cases with multiple congenital and/or chromosomal anomalies.

Optic nerves can be included in the imaging, but clinical examination is more sensitive in detecting optic nerve hypoplasia.

#### Ophthalmological examination

Infants with congenital CNS malformations should undergo routine ophthalmological examination to assess for associated eye pathology. This could include microphthalmia or frank globe malformation associated with dystroglycanopathies, colobomas, cataracts such as associated with COL4A1 mutations, chorioretinal lacunae suggestive of Aicardi syndrome, or chorioretinal scarring suggestive of a TORCH infection.

#### EEG

EEG, either conventional or amplitude integrated, should be obtained if seizure is considered. It is often extremely difficult to differentiate a seizure from myoclonus or other benign rhythmic movements in a neonate without an EEG. Overtreatment should be avoided, given concerns about possible neurotoxic effects of some first line anti-epileptic medications, such as phenobarbital, in neonates.

#### Echocardiogram

Echocardiogram should be considered when clinically indicated based on hemodynamic parameters, or in the case of a syndrome known to be associated with congenital heart disease or when multiple congenital anomalies are present. Even with syndromes such as lissencephaly that are thought to be CNS-specific, there is a low incidence of associated structural heart anomalies. Conversely, the prevalence of structural brain anomalies in patients with congenital heart disease is 28%, typically ventriculomegaly, agenesis of the corpus callosum, ventricular bleeding, increased extra-axial spaces, vermian hypoplasia, white matter abnormalities and delayed brain development [29].

#### Abdominal ultrasound

Abdominal ultrasound should be considered when clinically indicated or in the case of a syndrome known to be associated with hepatic or renal anomalies or when multiple congenital anomalies are present.

#### Skeletal survey

Skeletal survey should be considered when there is evidence of a skeletal dysplasia or multiple congenital anomalies.

#### Screening laboratories

Neonates with midline defects, including holoprosencephaly, agenesis of the corpus callosum and septo-optic dysplasia are at risk for pituitary hormone deficiencies. The presence and location of the pituitary gland should be noted on postnatal imaging and the involvement of the optic nerves assessed. Optic nerve hypoplasia (ONH) is an independent risk factor for hypothalamic-pituitary dysfunction, with absence of the septum pellucidum having no prognostic value. The presence of ONH alone has a 60-80% risk of associated hypothalamic-pituitary dysfunction [30]. Growth hormone deficiency is the most common deficiency in children with ONH, either presenting alone or in combination with other deficiencies. Posterior hormone deficiency resulting in diabetes insipidus is relatively uncommon, although can occur [31]. In one case series of infants with central diabetes insipidus, 5/19 patients had a primary diagnosis of SOD and an additional 4 were identified as having HPE. All of the SOD patients had other hormone deficiencies [32].

In addition to the possibility of hypothyroidism, neonates may experience hypoglycemia related to cortisol or GH deficiency. Screening laboratories might include a morning BMP for glucose and sodium, TSH, free T4, cortisol, IGF-1, IGFBP-3 and urine specific gravity. Any borderline or frankly abnormal values should prompt a formal consultation with a pediatric endocrinologist for further evaluation and management.

Infectious screening: given the potential association between TORCH infections, screening laboratories can be considered, in particular, urine CMV testing for suspected cases of congenital CMV, which might present with microcephaly, polymicrogyria and white matter abnormalities, or Zika testing, in the case of an appropriate travel history. HSV testing should also be considered, as wells as toxoplasmosis, especially with suspicious ocular findings.

Metabolic testing: A number of inborn errors of metabolism have been associated with brain malformations [33]. For example, peroxisomal disorders, such as Zellweger spectrum disorders, are often associated with polymicrogyria. Appropriate screening for this class of disorders would include very long chain fatty acids. Nonketotic hyperglycinemia can be associated with agenesis of the corpus callosum and would be diagnosed by comparing the ratio of glycine between serum and CSF plasma amino acids.

Genetic testing: Genetic counseling should be offered prenatally, which would include discussion of diagnostic testing. Amniotic fluid collection could be performed after 15 weeks *in utero* with minimal risks to pregnancy. Amniotic fluid could be sent for evaluation of infections, antigen testing, or genetic studies (karyotype, microarray or whole exome sequencing). Alternatively, cord blood should routinely be obtained when a CNS malformation is suspected prenatally. If not performed prenatally following amniocentesis, chromosomal microarray is often the first line genetic testing for many of these disorders. Otherwise, targeted gene panel testing or whole exome sequencing may be considered.

### Additional considerations

Apnea monitoring: With significant malformations of cortical development, there is a risk of brainstem dysfunction, which may present as central apnea, or the periodic breathing associated with JSRD. Infants should undergo routine cardiorespiratory monitoring until this concern is allayed or appropriate respiratory supports are put into place.

Feeding: Infants with congenital brain malformations not uncommonly have issues with coordination of suck and swallow and should be assessed clinically for their ability to feed safely, with formal evaluation if warranted.

A child neurologist should be consulted whenever a seizure is diagnosed or for infants at high risk for seizures based on cortical CNS involvement, or whenever the exact etiology of the CNS malformation if unknown.

Neurosurgical consultation should be obtained when there is a risk of, or frank evidence of hydrocephalus. A number of brain malformations can be associated with the need for surgical management, such as potentially fenestration of the third ventricle for cases of aqueductal stenosis or CSF shunting. Head circumferences should be routinely monitored, in addition to anterior fontanelle fullness and any clinical signs suggestive of increased intracranial pressure, with judicious use of routine head ultrasounds and/or ultrafast MRI to follow ventricular size.

Treatment of seizures: When seizures are present, they are typically treated with routine anticonvulsant agents.

Physical therapy and occupational consultations and post-discharge neurodevelopmental follow up should be considered.

Management recommendations are summarized in the following table: Summary of immediate postnatal recommendations:

- General and neurological examination
- Brain MRI
- Serial head circumference measurements
- Cardiorespiratory monitoring
- Feeding evaluation
- Ophthalmological exam
- Diagnostic work up: metabolic studies, genetic testing

Consideration of:

- Spine imaging (via ultrasound or MRI)
- Laboratory screening studies
- Echocardiogram
- Abdominal ultrasound
- Skeletal survey
- EEG

## Data Availability

Not applicable.
